# Increased susceptibility to organic dust exposure-induced inflammatory lung disease with enhanced rheumatoid arthritis-associated autoantigen expression in HLA-DR4 transgenic mice

**DOI:** 10.1186/s12931-022-02085-8

**Published:** 2022-06-18

**Authors:** Jill A. Poole, Ted R. Mikuls, Geoffrey M. Thiele, Rohit Gaurav, Amy J. Nelson, Michael J. Duryee, Ananya Mitra, Carlos Hunter, Todd A. Wyatt, Bryant R. England, Dana P. Ascherman

**Affiliations:** 1 Department of Internal Medicine, College of Medicine, University of Nebraska Medical Center, Omaha, NE USA; 2 Veterans Affairs Nebraska-Western Iowa Health Care System, Research Service, Omaha, NE USA; 3 Department of Environmental, Agricultural & Occupational Health, College of Public Health, University of Nebraska Medical Center, Omaha, NE USA; 4 Department of Medicine, School of Medicine, University of Pittsburgh, Pittsburgh, PA USA

## Abstract

Immunogenetic as well as environmental and occupational exposures have been linked to the development of rheumatoid arthritis (RA), RA-associated lung disease, and other primary lung disorders. Importantly, various inhalants can trigger post-translational protein modifications, resulting in lung autoantigen expression capable of stimulating pro-inflammatory and/or pro-fibrotic immune responses. To further elucidate gene-environment interactions contributing to pathologic lung inflammation, we exploited an established model of organic dust extract (ODE) exposure with and without collagen-induced arthritis (CIA) in C57BL/6 wild type (WT) versus HLA-DR4 transgenic mice. ODE-induced airway infiltration driven by neutrophils was significantly increased in DR4 versus WT mice, with corresponding increases in bronchoalveolar lavage fluid (BALF) levels of TNF-⍺, IL-6, and IL-33. Lung histopathology demonstrated increased number of ectopic lymphoid aggregates comprised of T and B cells following ODE exposure in DR4 mice. ODE also induced citrullination, malondialdehyde acetaldehyde (MAA) modification, and vimentin expression that co-localized with MAA and was enhanced in DR4 mice. Serum and BALF anti-MAA antibodies were strikingly increased in ODE-treated DR4 mice. Coupling ODE exposure with Type II collagen immunization (CIA) resulted in similarly augmented pro-inflammatory lung profiles in DR4 mice (relative to WT mice) that was accompanied by a profound increase in infiltrating lung CD4^+^ and CD8^+^ T cells as well as CD19^+^CD11b^+^ autoimmune B cells. Neither modeling strategy induced significant arthritis. These findings support a model in which environmental insults trigger enhanced post-translational protein modification and lung inflammation sharing immunopathological features with RA-associated lung disease in the selected immunogenetic background of HLA-DR4 mice.

**Supplementary Information:**

The online version contains supplementary material available at 10.1186/s12931-022-02085-8.

## Introduction

Although the primary manifestations in rheumatoid arthritis (RA) relate to inflammatory arthritis, respiratory-related deaths are the most over-represented cause of death in men and women with RA [1–3]. Multiple genetic and environmental risk factors contribute to the development of RA and RA-associated lung disease. These risk factors include the “shared epitope,” specific peptide sequences in selected type II human leukocyte antigens (HLA) such as HLA-DRB1*0401 (DR4) [4, 5]. Whereas the association among DR4 alleles and autoimmunity is well-recognized, it is not fully understood how this shared epitope predisposes an individual to autoimmune disease. It is thought that these polymorphisms allow for varying and concentrated peptide binding specificity to selectively stimulate self-reactive CD4^+^ T cells [6]. Extra-articular manifestations of RA are more common in individuals possessing the DR4 haplotype [7], yet the link between DR4 and RA-associated lung disease is less clear. While several studies demonstrate increases in the prevalence of interstitial lung disease (ILD) and diffuse pan-bronchiolitis in RA patients with DR4 [8–10], others have shown that DR4 exerts a protective effect against RA-ILD [10]. Beyond RA, the HLA-DRB1 alleles strongly influence the risk of developing sarcoidosis subtypes including Löfgren’s syndrome (acute clinical presentation of systemic sarcoid) [11–13]; in addition, there have been consistent associations of DR4 with idiopathic bronchiectasis as well as obliterative bronchiolitis [14, 15]. Collectively, these studies support a role for DR4 in inflammatory lung diseases, but the mechanisms underlying these associations and the specific contribution of DR4 to RA-associated lung disease remains unknown.

Various airborne inflammatory exposures are recognized as shared risk factors for primary lung diseases (e.g., chronic obstructive pulmonary disease, bronchitis, asthma) and for RA and RA-associated lung disease [3, 5, 16–19]. The risk associated with these exposures appears to be disproportionately higher in men, even though RA is more common among women [16]. Relevant exposures include not only cigarette smoke [3, 5], but also other exposures related to ambient air pollution, military service (burn pits, organic dusts, military waste disposal), and various workplace environments (agriculture organic dusts, textiles, silica, construction, mining) [3, 5, 16–18, 20]. The importance of environmental insults is further highlighted by emerging evidence linking the generation of autoantigens and RA-specific autoimmunity to an initial “breach” of tolerance in the respiratory mucosa [21–25]. Importantly, gene-environment interactions related to these environmental exposures likely impact disease susceptibility. For example, there is increased severity of lung silicosis in persons with HLA-DR4 [26]. Moreover, a history of military burn pit and waste exposures in veterans with RA and the shared epitope has been linked to serum RA autoantibody levels [5]. In contrast to these epidemiologic findings, experimental models have demonstrated that cigarette smoke can suppress collagen-induced arthritis (CIA) development in HLA-DR4 transgenic (Tg) mice, despite heightened post-translational modification (citrullination) and vimentin expression in bronchoalveolar lavage fluid of mice treated with cigarette smoke constituents [27].

Additional animal models have investigated mechanisms of RA-associated lung disease through strategies employing exposures to airborne inflammatory agents in the context of collagen-induced arthritis (CIA) [28]. These studies have demonstrated that coupling repetitive exposure to agriculture-related organic dust extracts (complex mixtures comprised of an abundance of bacterial products and particulates) with Type II collagen immunization (CIA) in the arthritis- susceptible DBA/1 J murine strain potentiated arthritis, expression of post-translationally modified autoantigens (including both citrullinated (CIT)- and malondialdehyde acetaldehyde (MAA)-modified lung proteins), formation of anti-MAA autoantibodies, and pro-fibrotic lung indices [29]. Importantly, sex differences that have been reported in epidemiological investigations [16] were recapitulated in this pre-clinical mouse model, as male mice were profoundly more susceptible than female mice to most effects of inhalant organic dust extract and CIA co-exposure. We hypothesized here that having an immunogenetic risk allele such as HLA-DR4 would potentiate environmental exposure-induced lung disease through development of post-translation protein modification, autoantibody production, and a proinflammatory/profibrotic phenotype characteristic of RA-associated lung disease to underscore the importance of gene-environment interactions.

## Materials and methods

### Mice

The generation of the HLA-DRB1*0401 (DR4)-transgenic (Tg) mice on C57BL/6 to provoke susceptibility of autoimmune disease has been previously described [30]. These mice were purchased with C57BL/6 control (or wild type, WT) mice from Taconic Biosciences (Germantown, NY). Male mice were utilized for all studies because we have previously demonstrated that female DBA/1J mice are strikingly less susceptible to organic dust extract (ODE)-associated lung disease, autoimmunity, arthritis, and periarticular bone loss in the setting of arthritis induction [29]. Mice between 6 and 8 weeks of age were allowed to acclimate for 1 week prior to initiation of experiments. Airway inflammation was induced using an established intranasal inhalation repetitive ODE exposure animal model [31] whereby mice were lightly sedated under isoflurane and received daily treatment with either 50 µL of sterile saline or 12.5% ODE daily for 4–5 weeks (weekends excluded). The collagen induced arthritis (CIA) model was also used in experimental studies per the Chondrex protocol (Chondrex, Inc, Redmond, WA) [29]. Briefly, 2 mg/mL of chick type II collagen emulsified in Freund's complete adjuvant was injected subcutaneously on day 1 at a final concentration of 100 μg and again at Day 21, using chicken collagen emulsified in Freund's incomplete adjuvant. Animals were euthanized 5 h following the final inhalant exposure. Animal procedures were approved by the Institutional Animal Care and Use Committee and were in accordance with the NIH guidelines for the use of rodents.

### Organic dust extract

Aqueous ODE was prepared from swine confinement feeding facilities as previously described [29]. Briefly, settled surface dust (1 g) was incubated in sterile Hank’s Balanced Salt Solution (10 mL; Mediatech, Manassas, VA) for 1 h and centrifuged for 30 min at 2850*g* and repeated, with the final supernatant filter-sterilized (0.22 um) to remove microorganisms and coarse particles. Endotoxin concentrations of 100% ODE ranged from 1200 to 1400 EU/mL as determined using the limulus amebocyte lysate assay (Lonza, Walkersville, MD). Muramic acid levels (bacterial cell wall peptidoglycans) were previously determined by mass spectrometry to be approximately 70 ng/mg [32]. Stock ODE was batch prepared and stored at − 20 °C; aliquots were diluted for each experiment to a final concentration (vol/vol) of 12.5% in sterile phosphate buffered saline (PBS, pH 7.4; diluent). ODE 12.5% has been shown to elicit optimal experimental outcomes and is well tolerated [29, 33].

### Cytokine, chemokine and fibronectin analysis

Bronchoalveolar lavage fluid (BALF) was collected using 3 × 1 mL PBS. Total cell numbers from the combined recovered lavage were enumerated and differential cell counts determined from cytospin-prepared slides (cytopro cytocentrifuge, ELITech Group, Logan, UT) stained with DiffQuick (Siemens, Newark, DE). From cell-free supernatant of the first lavage fraction, tumor necrosis factor-alpha (TNF-α), interleukin-6 (IL-6) and murine neutrophil chemokine (CXCL1) were quantitated by ELISA (R&D Systems, Minneapolis, MN; minimal detectable dose (MDD) of 7.2, 1.8, and 2.0 pg/mL, respectively). Lung tissue homogenates were prepared by homogenizing ½ right lung samples (half of each right cranial, middle, caudal, and accessory lobes) in 500 μl of sterile PBS. Lung tissue concentrations of fibronectin (Abcam, Cambridge, MA; MMD of 3 ng/mL) and the alarmin IL-33 (R&D; MMD of 14.3 pg/mL) were quantified by ELISA as previously described [34]. These analytes have been implicated in airborne biohazard-induced lung inflammation and RA-associated lung disease [29, 34].

### Lung histopathology

Following lavage, left lungs were excised and inflated to 15 cm H_2_O pressure with 10% formalin (Fisher Scientific, Fair Lawn, NJ) for 24 h to preserve pulmonary architecture as previously described [29]. The fixed left lung lobes were then placed into cassettes ventral side down, embedded in paraffin, cut (4–5 μM) at midpoint sections to include regions of both large and small airways as well as blood vessels, and stained with hematoxylin and eosin (H&E) (or preserved for subsequent IHC) by the Tissue Science Core Facility at the Department of Pathology and Microbiology at the University of Nebraska Medical Center. H&E-stained slides of the entire lung section from each animal were reviewed, and the number of ectopic lymphoid aggregates was assessed by a reviewer blinded to the treatment conditions.

To determine B cells, T cells, and neutrophils, lung sections were stained with anti-CD45R (1:100, Cat#14-0452-82, Lot#2178350; Invitrogen, Grand Island, NY), anti-CD3 (1:100, Cat#ab5690, Lot#GR3356033-2; Abcam) and anti-myeloperoxidase (MPO, 1:100, Cat#ab9535, Lot#GR331736-4; Abcam) antibodies. Cross absorbed (H + L) goat anti-rabbit Alexa Fluor (AF) 488 (Cat#A32731, Lot#UK290266), goat anti-mouse AF 555 (Cat#A32727, Lot#UL287768) and goat-anti rat AF 555 (Cat#A21434, Lot#2184321) from Thermo Fisher, Waltham, MA were used at 1:100 dilution as secondary antibodies. Slides were mounted with VECTASHIELD® Antifade Mounting Medium with DAPI (4′,6-diamidino-2-phenylindole; to identify nuclei) (Cat#H-1200, Lot#ZG1014, Burlingame, CA) and visualized under Zeiss fluorescent microscope. MPO^+^ neutrophils were quantified by Image J FIJI plug in as previously described [35].

### Lung autoantigens

Paraffin embedded lung sections from mice were rehydrated and subjected to antigen retrieval using citrate buffer and steam, and then blocked with goat serum and incubated with either Cy5 rabbit anti-vimentin (Bioss, Woburn, MA, 1:100), Zenon AF 594 label (Invitrogen, Carlsbad, CA), rabbit polyclonal IgG antibody to MAA, or a mouse monoclonal anti-peptidyl-citrulline antibody (clone F95 IgMκ, Millipore Sigma, Burlington, MA). Detection of the F95 antibody was done using an AF 488 AffiniPure donkey anti-mouse IgM, µ chain specific antibody (Jackson Immunoresearch, West Grove, PA). DAPI (4′,6-diamidino-2-phenylindole; to identify nuclei) was added and samples were sealed with Fluormount-G (Southern Biotech, Birmingham, AL). Fluorochrome detections were done using a Revolve fluorescent microscope (ECHO, San Diego, CA). Images were quantified using ImageJ (NIH, Bethesda, MD), and colocalization was performed using the Image J FIJI plugin Coloc 2 as previously described [34, 36].

### Serum pentraxin and autoantibodies

Serum was derived from whole blood collected from the axillary artery at the time of euthanasia [29]. Serum levels of Pentraxin-2 (murine acute phase reactant protein) were assessed by Quantikine ELISA according to the manufacturer’s instructions (R&D, MDD of 0.368 ng/mL). Anti-citrullinated protein antibody (ACPA) and anti-MAA antibodies (IgG) were quantified using an ELISA with data presented as relative units (RUs) of the specific isotype detected in the assay, as previously described [37, 38]. In brief, human serum albumin (HSA), type II collagen, and vimentin were used as substrate antigens for anti-MAA antibody measurement while HSA was used as the substrate antigen for ACPA quantification.

### Lung cell staining and flow cytometry

Following vascular perfusion and BALF removal, harvested lungs (right lungs) were subjected to an automated dissociation procedure using a gentle MACS Dissociator instrument (Miltenyi Biotech, Auburn, CA) [29]. Lung cells from each animal were incubated with a LIVE/DEAD Fixable Blue Dead Cell Stain Kit (Invitrogen, Carlsbad, CA) and CD16/32 (Fc Block, Biolegend) to minimize non-specific antibody staining. Cells were stained with monoclonal antibody (mAb) against rat anti-mouse CD45 BV605 (clone: 30-F11; BD Biosciences), CD11b peridinin-chlorophyll-protein (PerCP) Cy5.5 (clone: M1/70; BD Biosciences), Ly6G AF700 (clone 1A8; BD Biosciences), CD11c phycoerythrin (PE) eFluor 610 (clone: N418; Invitrogen), CD4 fluorescein-5-isothiocyanate (FITC) (clone RM4-5, BD Biosciences), CD8a PE (clone 53–6.7, BD Biosciences), CD19 allophycocyanin (APC) eFluor 780 (clone 1D3, Invitrogen), and hamster anti-mouse CD3e PE-Cy7 (clone 145-2C11, BD Biosciences). Gating strategies for Ly6G^+^ neutrophils, CD3^+^CD4^+^ T cells, CD3^+^CD8^+^ T cells, CD19^+^CD11b^−^ B cells (B1 B cells), and CD19^+^CD11b^+^ B cells (B2 B cells) (depicted in Additional file 1: Fig. S1); and for CD11c^+^CD11b^lo^ alveolar macrophages, CD11c^+^CD11b^hi^ activated macrophages, CD11c^int^CD11b^hi^ transitioning monocytes-macrophages, and CD11c^−^CD11b^hi^ monocytes (depicted in Additional file 2: Fig. S2) were as previously reported [29, 34, 39, 40]. The percentage of all respective cell populations was determined from live CD45^+^ lung leukocytes after excluding debris and doublets. This percentage was multiplied by the respective total lung cell numbers to determine specific cell population numbers for each animal.

### Arthritis evaluation

Mice were assessed weekly for the development of arthritis using the semi-quantitative mouse arthritis scoring system provided by Chondrex (www.chondrex.com). This protocol is based on hind-foot examination with a range of 0 (no inflammation) to 4 (erythema and severe swelling encompassing ankle, foot, and digits).

### Statistical analysis

Data are presented as the mean with standard deviation bars. To detect significant changes within group between saline and ODE, Mann–Whitney test was used. To detect interaction between genetic modification (DR4 and WT) and environment (saline and ODE), we used a two-way analysis of variance (ANOVA) to obtain P-interaction values. In the second set of studies, a one-way ANOVA with Tukey *post-hoc* test for multiple comparisons was utilized. In confocal studies, the FIJI plugin, Coloc 2 in ImageJ was used to quantify the magnitude of co-localization of vimentin, MAA, or CIT using five regions of interest from each image. Pearson’s correlation coefficients were calculated based on the overlap of two colors to generate an R^2^-value. R^2^-values from the five regions of interest were averaged for each mouse and compared using a one-way ANOVA with Tukey’s post hoc test to account for multiple comparisons. All statistical analyses were performed using GraphPad Prism (version: 9.0.0) software and statistical significance accepted at a two-sided p < 0.05.

## Results

### Increased airway inflammatory response to ODE in DR4 mice

**Fig. 1:**
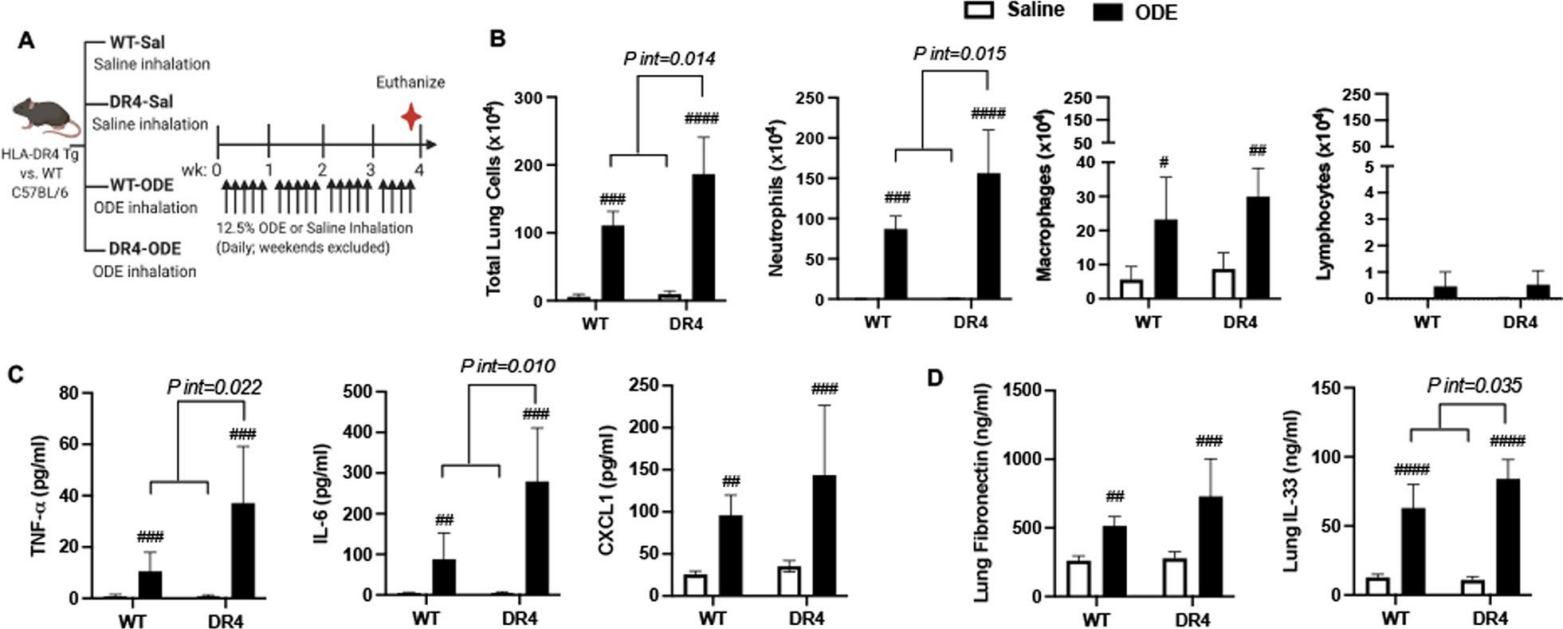
ODE-induced airway inflammatory response is increased in HLA-DR-transgenic mice. **A** Schematic of experimental design; Tg = Transgenic. Bar graphs in panels **B**–**D** depict mean + SD of cells and cytokines/chemokines from bronchoalveolar lavage fluid (BALF) and lung homogenates corresponding to indicated treatment groups. **B** BALF total cells, neutrophils, macrophages, and lymphocytes are enumerated. **C** BALF levels of TNF-⍺, IL-6, and CXCL1 and **D** lung levels of fibronectin and IL-33 determined by ELISA. N = 5 mice/group. Statistical differences versus saline treated mice are depicted by: ^#^p < 0.05, ^##^p < 0.01, ^###^p < 0.001, ^####^p < 0.0001. Brackets between groups represents significant gene-ODE interaction (*P-interaction [int]*)

In the first set of experiments, HLA-DR4 transgenic (Tg) and WT mice underwent daily intranasal inhalation of saline or ODE for 4 weeks (weekends excluded) and were euthanized 5 h following final exposure (schematic, Fig. 1A). ODE exposure induced increases in BALF total mononuclear cells, neutrophils, and macrophages without significant changes in lymphocytes (Fig. 1B). Eosinophils were rare and did not differ across groups (data not shown). There was significant DR4 (gene)-ODE interaction (*int*) for total cellular and neutrophil influx as well as levels of TNF-⍺, IL-6, and IL-33 (Fig. 1B–D). As compared to WT mice, total cellular influx (*P int* = 0.014) induced by ODE was increased in the DR4 mice, and this difference was driven predominantly by neutrophil influx (+ 1.8-fold increase, *P int* = 0.015). Correspondingly, ODE exposure resulted in an increase in BALF levels of airway pro-inflammatory cytokines (TNF-⍺, IL-6) and murine neutrophil chemoattractant (CXCL1) in DR4 mice, with potentiated levels of TNF-⍺ (+ 3.5-fold increase *P int* = 0.022) and IL-6 (+ 3.4-fold increase, *P int* = 0.010) relative to WT mice (Fig. 1C). ODE also induced increased lung tissue levels of fibronectin and IL-33 in both WT and DR4 mice, with IL-33 levels (but not fibronectin) being significantly increased in DR4 compared to WT animals (*P int* = 0.035) (Fig. 1D).

### ODE-induced ectopic lymphoid aggregates and neutrophil infiltrates are increased in the lungs of DR4 mice

**Fig. 2:**
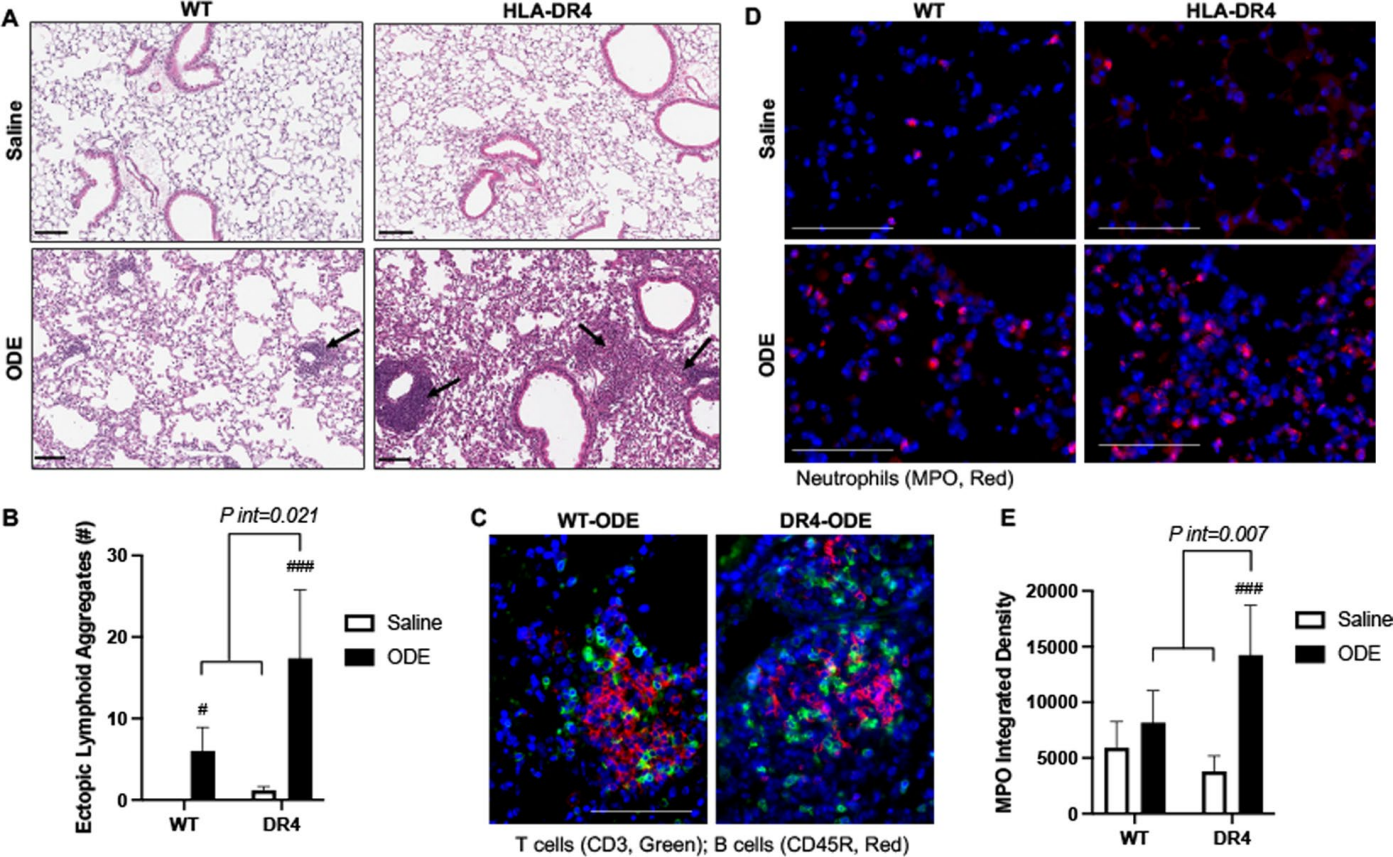
ODE-induced ectopic lymphoid aggregates and neutrophil infiltrates are increased in DR4 mice. **A** Representative H&E-stained lung section images from each treatment group with arrows indicating ectopic lymphoid aggregates. **B** Bar graph (mean + SD) demonstrating the number of aggregates enumerated in different treatment groups. **C** Confocal microscopy image of T cells (green), B cells (red), and nuclei (DAPI, blue) within ODE-induced ectopic lymphoid aggregates in WT and DR4 mice. **D** Confocal microscopy images of myeloperoxidase (MPO, red) staining of neutrophils and DAPI (blue) in lung parenchyma from designated treatment groups. **E** Bar graphs showing integrated density of MPO^+^ neutrophil staining. N = 5 mice/group. Statistical differences versus saline control group are indicated by ^##^p < 0.01, ^###p^ < 0.001. Brackets between groups represents significant gene-ODE interaction (*P-int*). Line scale is 100 μm

Repetitive ODE exposure induces ectopic lymphoid aggregates [29, 33], and in DR4 mice, ODE-induced lymphoid aggregates were markedly increased in number compared to WT (Fig. 2A, B). These lymphoid aggregates were comprised of both B and T cells (Fig. 2C), consistent with prior observations regarding ODE-induced pathology [33]. Moreover, ODE-induced neutrophil infiltrates in the lung parenchyma were significantly increased in DR4 relative to WT mice (Fig. 2D, E). There was significant DR4-ODE interaction for both aggregate number (*P int* = 0.021) and lung tissue neutrophil infiltrates (*P int* = 0.007). Collectively, these studies support an enhanced airway inflammatory and pathologic response following repetitive ODE exposure in DR4 mice.

### ODE induces MAA and CIT post-translational modifications with increased vimentin expression and MAA-vimentin co-localization in the lungs of DR4 mice

**Fig. 3:**
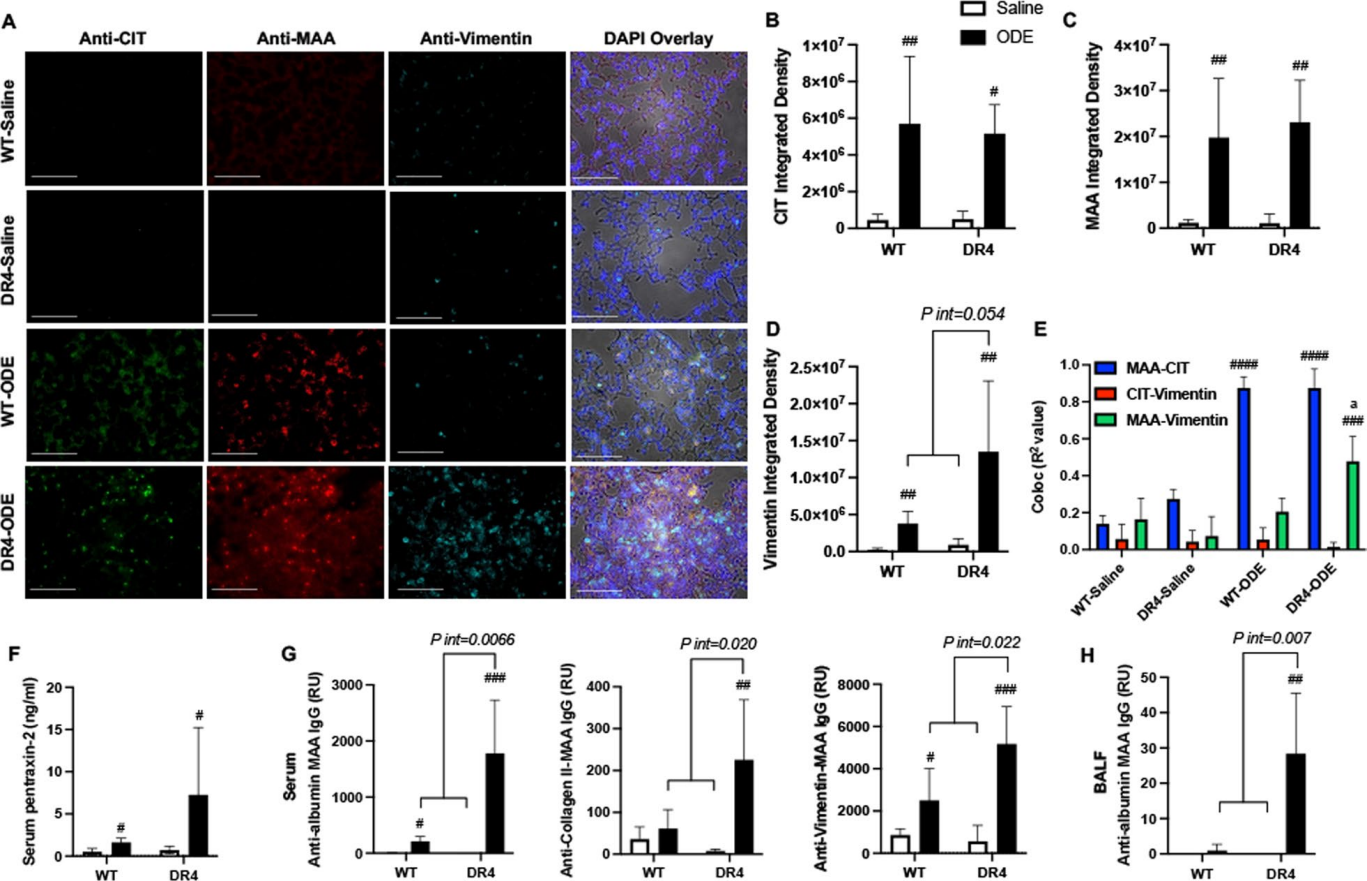
Expression of lung autoantigens, serum inflammatory markers, and autoantibodies following ODE exposure. **A** Confocal images of lung tissue from treatment groups stained for citrulline (CIT)- and malondialdehyde-acetaldehyde (MAA)-modified proteins as well as vimentin, with nuclei staining by DAPI (blue). Images were analyzed using Zen 2012 software (Zeiss). Bar graphs depict mean integrated density (+ SD) of **B** CIT- or **C** MAA-modified proteins, and **D** vimentin, quantified per each mouse. **E** R^2^-values demonstrating co-localization of MAA-CIT, CIT-Vimentin, and MAA-Vimentin within each treatment group. Panels **F**–**H** demonstrate serum levels of the murine acute phase reactant protein, pentraxin-2 (**F**), serum levels of IgG antibody to MAA-modified proteins utilizing human serum albumin, collagen type II, and vimentin as the substrate antigen (**G**), and BALF levels of IgG antibody to MAA (**H**), all quantified by ELISA. Statistical differences versus saline-treated mice are indicated by ^#^p < 0.05, ^##^p < 0.01, ^###^p < 0.001, ^####^p < 0.0001. Brackets between groups represents significant gene-ODE interaction (*P-int)* and “a” (p < 0.05) is WT-ODE vs. DR4-ODE. RU: relative units. Line scale is 100 μm

Lung tissues were stained and analyzed for CIT- and MAA-modified antigens that included vimentin. Vimentin is an extracellular matrix protein that is increased in inflammatory lung diseases such as RA-ILD [34, 41, 42] and can be targeted by post-translational protein modifications, as demonstrated by robust co-localization with MAA in RA-ILD lung tissues [41]. By confocal microscopy, CIT- and MAA-modified proteins as well as vimentin were significantly increased in WT-ODE and DR4-ODE as compared to respective saline controls (Fig. 3A–D). Whereas ODE-induced CIT- and MAA-modified antigens were similarly increased in WT and DR4 mice (Fig. 3B), ODE-induced vimentin expression was potentiated in DR4 mice compared to WT mice (+ 3.5-fold increase), but this did not meet interaction significance (P int = 0.054, Fig. 3D). Pearson correlation coefficients and R^2^ values were calculated as measures of autoantigen co-localization in lung tissues. CIT and MAA modifications strongly co-localized following ODE exposure in both WT mice (R^2^ value of 0.82) and DR4 mice (R^2^ value of 0.91) (Fig. 3E). However, MAA and vimentin co-localization was more pronounced in ODE-exposed DR4 mice (R^2^ value of 0.48) compared to ODE-exposed WT mice (R^2^ value of 0.21. p = 0.013). There was only minimal co-localization of CIT with vimentin.

### Increased ODE-induced anti-MAA autoantibodies and acute phase reactants in DR4 mice

Serum concentrations of the murine acute phase reactant protein, pentraxin-2, were increased with ODE exposure, and this response was increased in DR4 relative to WT mice (+ 4.5-fold increase, Fig. 3F). Serum anti-MAA IgG antibody responses utilizing HSA, type II collagen, and vimentin as substrate antigen(s) were strikingly enhanced in DR4 mice treated with ODE compared to mice receiving saline inhalation (Fig. 3G). In WT animals, there was also a significant increase in ODE-induced anti-albumin-MAA and anti-vimentin-MAA (but not anti-collagen II-MAA) IgG responses. Compared to WT animals, ODE exposure in DR4 mice significantly enhanced IgG responses to albumin-MAA (+ 8.4-fold increase, *P-int* = 0.0066), collagen II-MAA (+ 2.1-fold increase, *P-int* = 0.020), and vimentin-MAA (+ 3.7-fold increase, *P-int* = 0.022). In turn, there was a profound increase (~ 28-fold) in ODE-induced anti-MAA IgG antibodies measured in BAL fluid that was limited to DR4 animals (Fig. 3H), significantly increased (*P-int* = 0.007) compared to ODE-exposed WT mice. Serum ACPA responses, measured using citrullinated HSA as the substrate antigen, were not detected (data not shown).

### ODE exposure induces minimal evidence for arthritis in DR4 mice

Following 4 weeks of ODE exposure, there was only slight evidence of arthritis (arthritis inflammatory score, mean ± SD) in WT-ODE (0.2 ± 0.27; p = 0.17) and DR4-ODE (0.3 ± 0.27; p = 0.049) versus WT-saline (0.0 ± 0.0) and DR4-saline (0.0 ± 0.0) control mice, respectively (Additional file 4: Fig. S4A).

### Combined collagen-induced arthritis (CIA) and ODE exposure triggers enhanced airway inflammation in DR4 mice

The C57BL/6 murine strain is not commonly used for RA modeling due to its resistance in developing collagen-induced arthritis (CIA) [43, 44]. In contrast, the genetically disparate DBA/1J murine strain is typically used in CIA modeling studies [45]. Previously, we demonstrated that repetitive ODE exposure potentiates arthritis and systemic autoimmunity in DBA/1J mice [29]. However, ODE-induced inflammatory cell influx and cytokine/chemokine release were decreased (as opposed to increased) in the setting of concomitant CIA (i.e., CIA + ODE) in the airways of DBA/1J mice. Coupled with evidence of skewing towards a pro-fibrotic lung phenotype with CIA + ODE [29], these findings suggested that arthritis induction may modulate the relative balance between inflammation and fibrosis in the lungs.

**Fig. 4:**
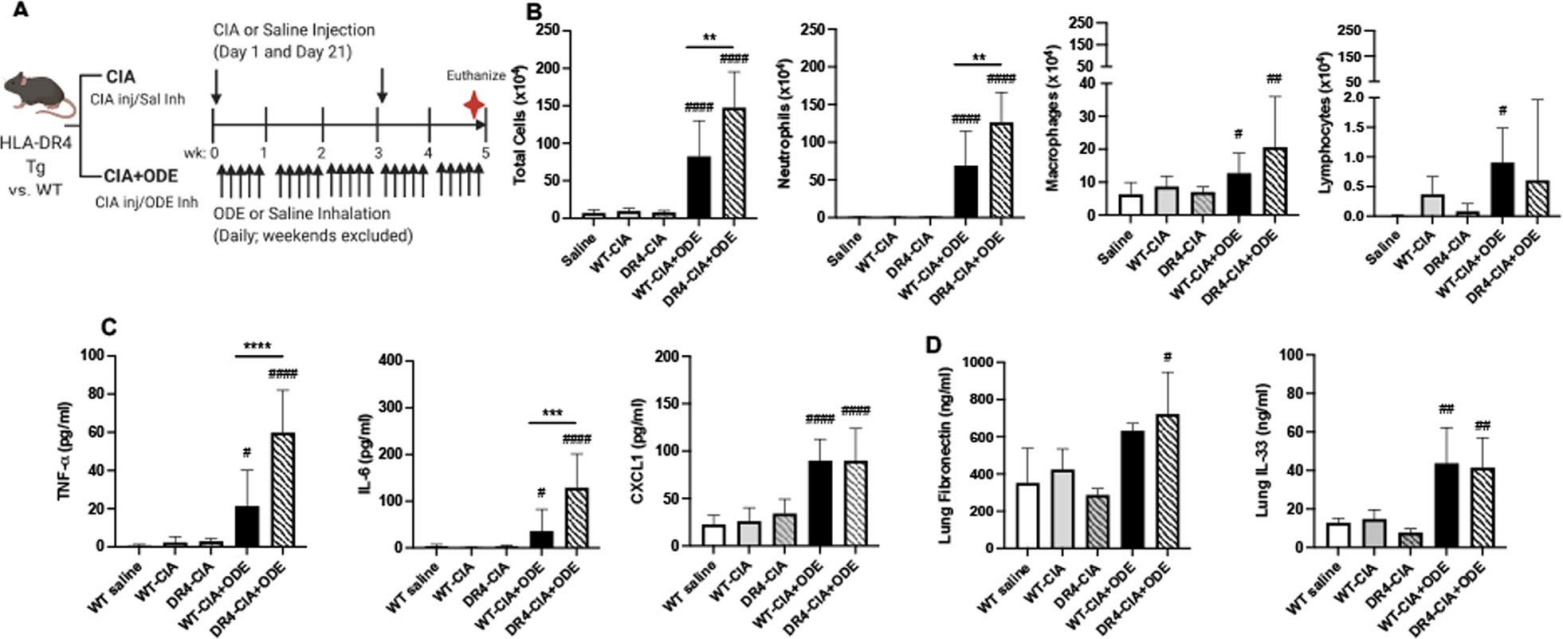
ODE-induced airway inflammatory response is increased in the setting of collagen-induced arthritis (CIA) in DR4 mice. **A** Schematic of experimental design. Bar graphs in **B**–**D** depict mean (+ SD) number of cells and levels of cytokines/chemokines from bronchoalveolar lavage fluid (BALF) and lung homogenates in designated treatment groups. **B** BALF total cells, neutrophils, macrophages, and lymphocytes are enumerated. **C** BALF levels of TNF-⍺, IL-6, and CXCL1 and **D** lung levels of fibronectin and IL-33, as determined by ELISA. N = 5–7 mice/group (BALF) and N = 3–5 (Lung). Statistical differences versus respective saline-treated mice are marked by ^#^p < 0.05, ^##^p < 0.01, ^####^p < 0.0001. Lines denote difference between groups (**p < 0.01, ***p < 0.001, ****p < 0.0001)

In the current studies, CIA modeling with and without ODE exposure (5 weeks) was applied to both C57BL/6-derived DR4 and WT mice (schematic, Fig. 4A). Replicating the data obtained without concomitant Type II collagen immunization (Fig. 1), ODE exposure induced increases in BALF total mononuclear cells, neutrophils, and macrophages in both WT-CIA + ODE and DR4-CIA + ODE animals compared to saline control mice (Fig. 4B). Again, the ODE-induced influx of total cells and neutrophils was significantly increased in DR4 mice compared to WT mice. As in the initial experiments with ODE alone, combined CIA + ODE exposure resulted in increased BALF levels of airway proinflammatory cytokines (TNF-⍺, IL-6) and murine neutrophil chemoattractant (CXCL1) in both DR4 and WT mice (Fig. 4C). Furthermore, CIA + ODE exposure potentiated levels of TNF-⍺ (+ 2.8-fold increase, p < 0.0001) and IL-6 (+ 3.6-fold increase, p = 0.0004) in DR4 relative to WT mice, again replicating findings from experiments involving repetitive ODE inhalation alone. Lung tissue concentrations of fibronectin and IL-33 were increased with CIA + ODE in both WT and DR4 mice, without significant differences between strains (p > 0.05, Fig. 4D). Of note, Type II Collagen immunization (CIA) alone did not induce airway cellular influx or inflammatory cytokine release in either WT or DR4 mice.

### ODE-induced lung infiltrates are increased in the setting of CIA in DR4 mice

**Fig. 5:**
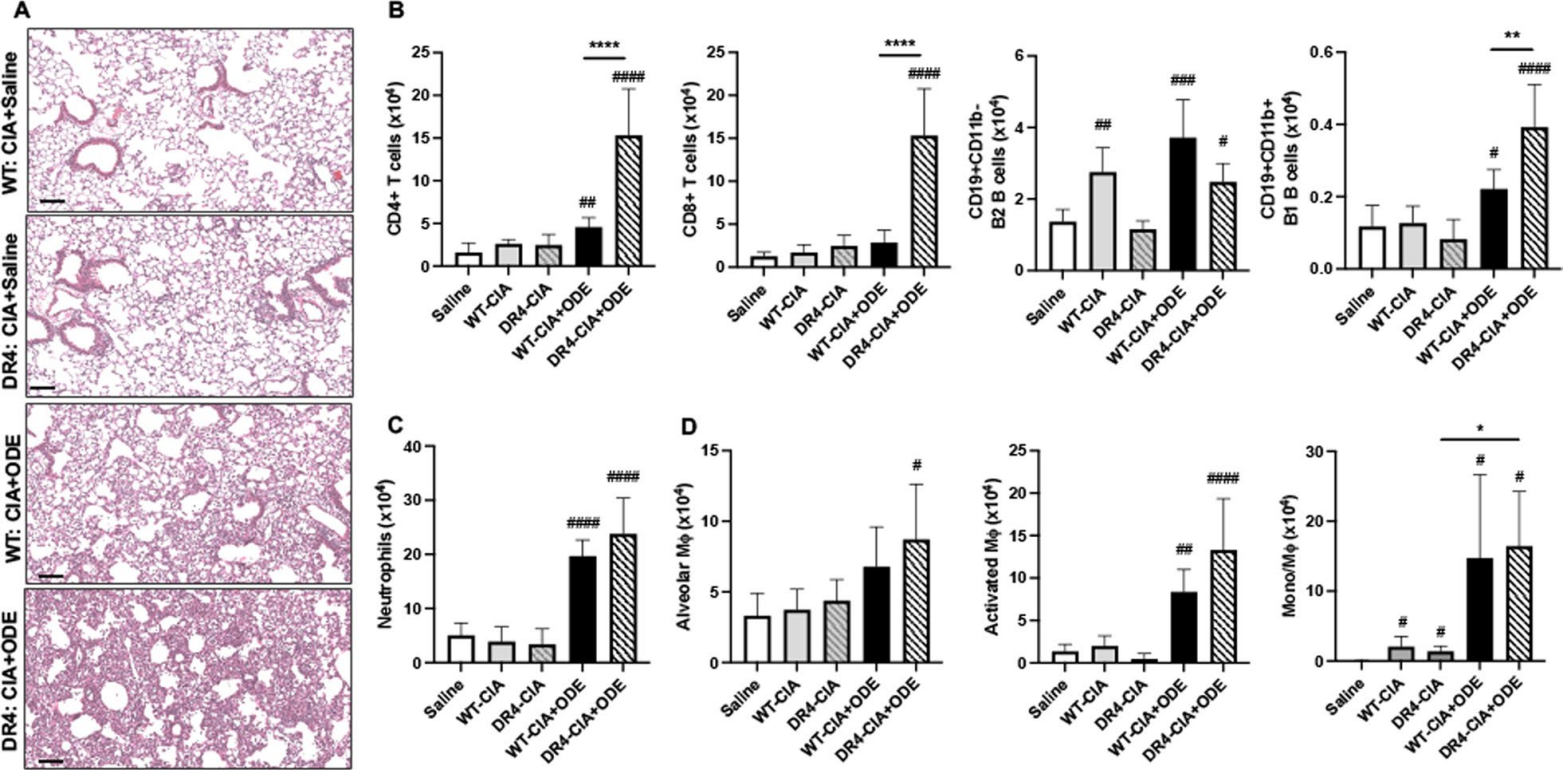
ODE-induced lung infiltrates are increased in the setting of CIA in DR4 mice. **A** Representative H&E-stained lung section images from each treatment group. **B**–**D** Lung cell infiltrates were determined by flow cytometry on live CD45^+^ cells after exclusion of debris and doublets. Number of cells was determined by multiplying the percentage of cell subtype by the total number of lung cells enumerated. **B** Lung lymphocytes defined as CD3^+^CD4^+^ T cells, CD3^+^CD8^+^ T cells, CD19^+^CD11b^−^ B2 B cells, and CD19^+^CD11b^+^ B1 B cell subpopulations. **C** Lung myeloid cells defined as CD11c^−^Ly6G^+^ neutrophils and **D** CD11c^+^CD11b^lo^ alveolar macrophages, CD11c^+^CD11b^hi^ activated macrophages, and CD11c^int^CD11b^hi^ transitioning monocytes-macrophages. Gating strategies are depicted in Additional file 1: Fig. S1, Additional file 2: Fig. S2. Statistical differences versus saline-treated mice are designated by ^#^p < 0.05, ^##^p < 0.01, ^###^p < 0.001, and ^####^p < 0.0001. Lines denote differences between groups (*p < 0.05, **p < 0.01, ****p < 0.0001). N = 5–7 mice/group. Line scale is 100 μm

Lung histopathologic changes consisting of cellular infiltrates and aggregates were most pronounced in DR4 mice treated with CIA + ODE compared to DR4 mice treated with CIA alone or to WT mice treated with CIA ± ODE (Fig. 5A). However, a limited number of scattered, small granulomas were observed in both WT and DR4 mice receiving CIA + saline.

Based upon studies demonstrating increased ectopic lymphoid aggregates in DR4 mice following ODE exposure (Fig. 2), lung tissues in the CIA + ODE modeling studies were processed for lymphocyte subsets, neutrophils, and monocyte/macrophage populations by flow cytometry (Fig. 2B–D). Lung-infiltrating CD4^+^ T cells were increased in WT-CIA + ODE mice, and this response was profoundly potentiated in the DR4-CIA + ODE mice (+ 3.4-fold increase, p < 0.0001). CD8^+^ T cells were also strikingly increased in DR4-CIA + ODE compared to WT-CIA + ODE mice (+ 5.4-fold increase, p < 0.0001), as neither CIA + ODE nor CIA alone led to increased CD8+ T cells in WT mice (relative to saline-treated control mice). B2 B cells (CD19^+^CD11b^−^), which are hallmark effectors of adaptive immune responses, were increased in WT-CIA, WT-CIA + ODE and DR4-CIA + ODE mice. B1 B cells (CD19^+^CD11b^+^) cells, which are implicated in innate defense against mucosal pathogens and autoimmunity development [39, 46], were increased in both WT-CIA + ODE and DR4-CIA + ODE mice, but most notably in DR4-CIA + ODE mice (+ 1.8-fold increase compared to WT-CIA + ODE, p < 0.01). Lung neutrophils were increased in both WT and DR4 mice treated with CIA + ODE (but not CIA alone), with no difference between groups (Fig. 5C). Resting alveolar macrophages (CD11c^+^CD11b^+^), activated macrophages (CD11c^+^CD11b^+^), and transitioning monocyte-macrophages (CD11c^int^CD11b^+^) were all increased in DR4-CIA + ODE mice, whereas only activated and transitioning monocyte/macrophage populations were increased in WT-CIA + ODE mice (Fig. 5D). Although these collective data indicated that the combination of CIA + ODE resulted in a proinflammatory lung phenotype that was particularly augmented in the DR4 immunogenetic background, there were increases in the transitioning monocyte/macrophage population in both WT and DR4 mice treated with CIA alone.

### Differential expression of ODE-induced induced lung autoantigens and vimentin in DR4 mice in the context of CIA

**Fig. 6:**
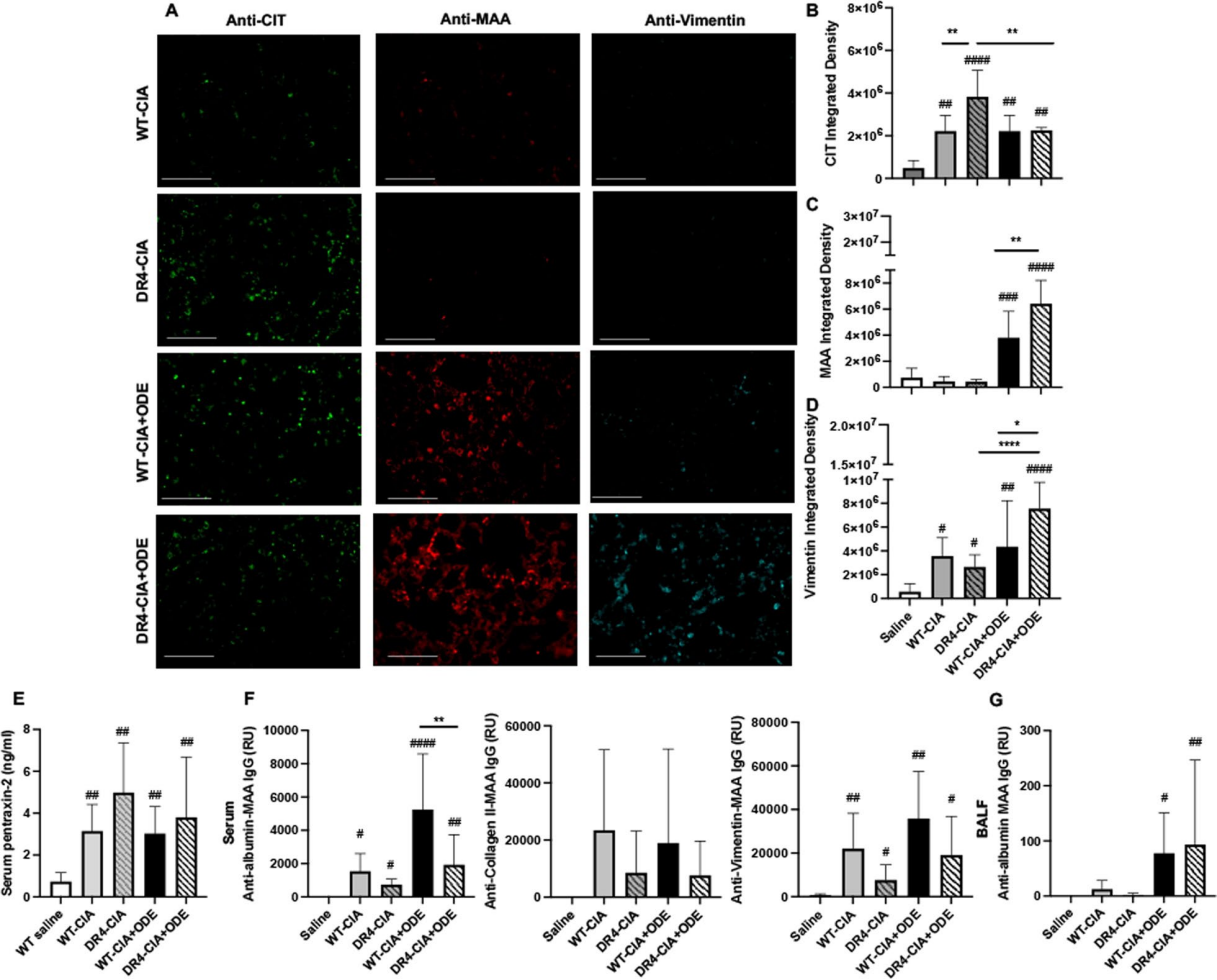
ODE-induced lung autoantigens, vimentin, serum inflammatory markers, and autoantibodies in the setting of CIA in DR4 mice. **A** Confocal images of lung tissue from treatment groups stained for citrulline (CIT)- and malondialdehyde-acetaldehyde (MAA)-modified proteins as well as vimentin. Bar graphs in **B**–**D** depict integrated density (+ SD) of **B** CIT- or **C** MAA-modified proteins, and **D** vimentin, quantified per each mouse. Bar graphs in **E**–**G** demonstrate serum levels of murine pentraxin-2 (**E**), serum levels of IgG antibody to MAA-modified proteins (**F**) utilizing human serum albumin, collagen type II, and vimentin as the substrate antigen, and BALF levels of IgG antibody to MAA (**G**), all quantified by ELISA. Statistical differences versus saline-treated control mice are designated by ^#^p < 0.05, ^##^p < 0.01, ^####^p < 0.0001. Lines denote differences between groups (*p < 0.05, **p < 0.01, ****p < 0.0001). RU: relative units. Line scale is 100 μm

Citrullinated (CIT) lung proteins were increased in WT and DR4 mice treated with CIA and CIA + ODE, with significantly increased CIT staining in the DR4-CIA animals compared to all other groups (Fig. 6A, B). In contrast, MAA expression was increased with CIA + ODE (but not CIA alone) in both WT and DR4 mice, with enhanced expression demonstrated in DR4 mice (+ 1.7-fold increase, p < 0.01) compared to WT animals (Fig. 6A and C). Likewise, there was an increase in vimentin expression with both CIA and CIA + ODE in WT and DR4 mice, but this response was significantly potentiated in DR4-CIA + ODE animals (+ 1.7-fold increase vs. WT-CIA + ODE, p = 0.02; Fig. 6A and D).

Paralleling the findings in experiments without CIA (Fig. 3), increased CIT- and MAA-modified proteins strongly co-localized in all treatment groups (Additional file 1: Fig. S4), with R^2^ values ranging from 0.82 to 0.88. MAA and vimentin also co-localized to a similar extent among different treatment groups, with R^2^ values ranging from 0.57 to 0.67. In contrast to findings with ODE alone (without CIA), CIT and vimentin co-localized to a similar degree in all mice subjected to CIA (± ODE), with R^2^ values ranging from 0.77 to 0.86. Importantly, however, vimentin was preferentially expressed in DR4 mice exposed to CIA + ODE, consistent with experiments involving ODE administration without Type II collagen immunization (Fig. 3).

### Combined CIA and ODE-induced systemic inflammation, autoantibodies, and arthritis in DR4 mice

Serum concentrations of the murine acute phase reactant protein, pentraxin-2, were increased among all treatment groups (WT-CIA, DR4-CIA, WT-CIA + ODE, and DR4-CIA + ODE) relative to saline-treated control mice, with no significant differences between treatment groups (Fig. 6E). Compared to models involving ODE exposure alone, the addition of CIA led to inconsistent serum autoantibody responses in WT versus DR4 mice (Fig. 6F). In WT animals, for example, there were increases in anti-albumin-MAA IgG antibodies with CIA and CIA + ODE treatment, whereas there were no increases in these antibodies in similarly treated DR4 mice. While none of the WT or DR4 treatment groups demonstrated increased anti-collagen II-MAA antibodies relative to saline treated control mice, anti-vimentin-MAA antibodies were increased in WT-CIA, WT-CIA + ODE and DR4-CIA + ODE mice. Anti-MAA IgG antibodies were also demonstrated in the BALF of both WT and DR4 animals treated with combined CIA + ODE, but there were no statistically significant differences between these two groups (Fig. 6G). Serum ACPA (anti-citHSA), on the other hand, were not detected among treatment groups.

Finally, there was only slight, but statistically significant, evidence to support arthritis induction (arthritis inflammatory score, mean ± SD) in WT-CIA + ODE (0.31 ± 0.26; p = 0.032) and DR4-CIA + ODE (0.40 ± 0.22; p = 0.014) mice versus saline-treated (0.0 ± 0.0) animals (Additional file 3: Fig. S3B).

## Discussion

Several shared genetic and environmental risk factors exist for both lung diseases and RA, with established and emerging evidence strongly supporting the concept that inflammatory and immune processes occurring in the lung microenvironment play a key pathologic role in the “pre-RA” period [47–49]. However, the mechanisms linking these complex processes to disease development, progression, and severity in genetically susceptible individuals remain undefined [49]. In general, post-translational protein modifications including citrullination and MAA-modification have been presumed to initiate autoimmune responses to self-peptides through generation of cryptic/neo-epitopes that enhance autoreactive lymphocytes and/or potentiate loss of tolerance to lung antigens [47, 49, 50]. Consistent with this paradigm, our results indicate that the HLA-DRB1 shared epitope expressed in HLA-DR4 transgenic mice strikingly predisposed animals to increased ODE-induced airway inflammatory responses, formation of lung ectopic lymphoid aggregates, and vimentin expression co-localizing with MAA. These findings correspond to increased concentrations of serum and BALF anti-MAA autoantibodies following ODE exposure in DR4 animals. The additional stimulus of Type II collagen immunization (CIA) did not substantially change the pro-inflammatory lung profile of DR4 mice but did promote lung infiltration by CD4^+^ and CD8^+^ T cells in this strain.

Extensive genetic studies have demonstrated that the HLA-DRB1 shared epitope contributes to risk across different populations, particularly in ACPA-positive RA patients [49, 51]. The importance of these risk alleles is not limited to RA, as these HLA haplotypes are strongly associated with sarcoidosis (including Löfgren’s syndrome), obliterative bronchiolitis, and idiopathic bronchiectasis [11–15, 52]. Moreover, HLA-DRB1 alleles are linked to more severe forms of sarcoidosis, including a higher burden of lung granulomas as well as extrapulmonary manifestations such as hypercalcemia and ocular disease [52]. Our findings demonstrating significant gene (DR4) by environment (ODE) outcomes, strongly suggest that this unique MHC determinant markedly skews the host response to air pollutants towards a more robust lung inflammatory phenotype, with features shared by RA-associated lung disease as well as other primary inflammatory lung diseases. Given our experimental design, we were unable to formally test for gene-environment interactions in our studies incorporating CIA, which would require additional studies using larger sample sizes and additional treatment groups. Clinical and epidemiologic studies are warranted to evaluate whether assessment of HLA-DR status may provide prognostic or therapeutic guidance for patients suspected to have RA and/or inflammatory lung diseases. For example, studies have previously suggested that early and aggressive immunosuppressive treatment provides therapeutic benefit in seropositive RA patients possessing shared epitope DRB1 risk alleles [53, 54].

Respiratory-related disorders are the most overrepresented cause of death in RA [2, 55]. While symptomatic ILD occurs in up to 10–15% of RA patients and is associated with poor long-term prognosis [56–58], COPD remains the most common cause of respiratory related death in this patient population [2, 59, 60]. Various airborne environmental/occupational exposures (i.e., farming construction, mechanic, warehouse, and military work exposures) are not only risk factors for COPD but are also strongly associated with the risk of both RA and RA-associated lung disease [3, 5, 16–19]. Moreover, approximately 35% of agriculture workers develop airway inflammatory diseases including chronic bronchitis, COPD, and asthma, independently of RA [61, 62]. Prior murine studies have also established that agriculture-related organic dust extracts induce a potent neutrophilic and pro-inflammatory airway inflammatory response that lessens in magnitude over time, but results in the development of ectopic lymphoid aggregates consistent with a chronic inflammatory adaptive response [33]. In DR4 mice, this ODE-induced neutrophil and pro-inflammatory cytokine/chemokine responses were enhanced, resulting in particularly striking elevations of BALF TNF-α and IL-6 levels. These observations could have translational application for personalized medicine approaches, as they suggest that specific cytokine-targeted therapies may have improved efficacy in HLA-DRB1^+^ individuals with relevant occupational exposures and lung disease manifestations. Previous studies have, in fact, demonstrated that individuals with RA and specific HLA-DRB1 haplotypes have a more favorable response to TNF inhibitor drugs, at least for articular disease [63].

Beyond these inflammatory mediators, our studies have demonstrated that lung IL-33 levels are increased following airborne biohazard exposures, particularly in DR4 mice. As an alarmin that forms in response to damage or stress signals, IL-33 is increased in several inflammatory and fibrotic lung diseases that include COPD, idiopathic pulmonary fibrosis (IPF), post-COVID lung fibrosis, and RA-ILD [34, 42]. Given that clinical studies targeting the systemic IL-33 signaling pathway are currently underway in the treatment of COPD (clinicaltrials.gov; NCT04701983; NCT03615040), investigating the role of IL-33 in HLA-DRB1^+^ RA and lung diseases resulting from airborne exposures may be warranted.

MAA adducted proteins have been demonstrated in lungs of individuals with alcohol use disorder who smoke cigarettes [64]. Similarly, our results demonstrate that post-translational modifications encompassing CIT and MAA-modification were induced in lung tissue following organic dust exposure, with MAA and CIT strongly co-localizing. Our results are also consistent with previously reported animal studies using endotoxin exposure [34, 39] and a prior study from our group examining histopathologic lung tissues from patients with RA-associated lung disease [41]. Despite the overall increase in airway inflammation in DR4 mice, ODE-induced expression of lung CIT and MAA was similar in magnitude to that observed in WT animals (except in mice exposed to ODE + CIA, where MAA modification was augmented in DR4 relative to WT mice). However, ODE-induced vimentin expression was robustly increased in DR4 mice with or without Type II collagen co-immunization. Interestingly, MAA and vimentin co-localization was also more pronounced in DR4 mice. Vimentin appears to be a critical lung protein that is susceptible to post-translational modification and associated with disease activity. For example, administration of citrullinated vimentin promotes pulmonary fibrosis through its action as a Toll-like receptor 4 ligand [65].

Another striking finding in DR4 mice was the increase in number of ODE-induced ectopic lymphoid aggregates. The size of individual aggregates was variable and not formally assessed, potentially biasing the number of aggregates enumerated per lung section. Of note, additional lung phenotyping in the CIA + ODE model strongly supports an enhanced T-cell response with co-existing features of B cell autoimmunity. This enhanced T cell response supports previous studies showing that immunization of HLA-DR4 Tg mice with DR4-restricted peptides induces T cell proliferation in vitro [30]. Moreover, antigen presentation by B cells from DR4 Tg mice is enhanced in vitro and consistent with increased autoantibody development in these mice [66]—a phenomenon that we also observed in the current study. Complementing these findings in B and T cells, ex vivo stimulation of DR4 bone marrow-derived macrophages with various pro-inflammatory agents polarizes these cells toward an “M1” phenotype [67] that potentially overlaps with the activation profile of macrophages isolated from DR4 lung tissue following ODE inhalation. Collectively, the findings from our study and previous investigations indicate that DR4 likely mediates ODE-induced enhancement of lung-infiltrating T cells, autoimmune B cells, and activated macrophages.

Despite the increase in CIT- and MAA-modified lung proteins following ODE exposure, systemic disease manifestations and serum autoantibody responses were not entirely uniform between WT and DR4 mice. Systemic anti-MAA (but not anti-HSA citrullinated HSA) IgG antibody responses were much more pronounced in ODE-exposed DR4 mice, though this response was not enhanced (relative to WT mice) with concomitant administration of Type II collagen/CFA emulsions. Correspondingly, BALF anti-MAA IgG antibodies were demonstrated predominantly in the DR4 mice exposed to ODE alone (Fig. 3), while administration of CIA + ODE promoted the formation of anti-MAA IgG in the BALF of both WT and DR4 mice. In both ODE and CIA + ODE modeling systems, there was also a relative lack of arthritis development—regardless of immunogenetic strain background. Although the absence of arthritis and relative uncoupling from serum autoantibody formation was somewhat unexpected and represents a limitation to using this model in future arthritis-focused studies, there are several potential explanations for these observations. For example, generation of anti-MAA antibodies may reflect inflammatory consequences of ODE-induced insult of airway and lung tissue that heralds a “pre-RA” phase, but ultimately requires additional antigen-targeted immune responses to trigger development of arthritis. In fact, it is possible that arthritis might be generated over longer exposure periods, as has been reported in C57BL/6 mice undergoing an extended 3 month CIA protocol [43]. To enhance the arthritis phenotype, protocol modifications such as increasing the concentration of collagen immunization or immunization with citrullinated proteins may also be necessary. Even if immune responses targeting citrullinated antigens are ultimately required to develop the combined lung-joint phenotype of RA-ILD, however, anti-MAA antibodies may be useful biomarkers in pre-RA as well as other lung diseases that include sarcoidosis and idiopathic bronchiectasis.

In conclusion, environmental inflammatory insults such as complex organic dust extract trigger post-translational protein modifications and enhanced lung inflammation in the selected immunogenetic background of HLA-DR4 mice, producing an immunophenotype that shares pathological features with RA-ILD and other inflammatory lung diseases. These findings correspond to enhanced production of CIT- and MAA-modified lung autoantigens as well as generation of anti-MAA antibody (but not anti-HSA-CIT/ACPA), suggesting a potential role for MAA-targeted immune responses in the development of inflammatory lung disease with and without concomitant RA.

## Supplementary Information

**Additional file 1: Figure S1.** Flow cytometry gating strategies depicted for lung cell identification of Ly6G^+^ neutrophils, CD3^+^CD4^+^ T cells, CD3^+^CD8^+^ T cells, CD19^+^CD11b^−^ B2 B cells and CD19^+^CD11b^+^ B1 B cells.

**Additional file 2: Figure S2.** Flow cytometry gating strategy for lung macrophage-monocyte subpopulations is depicted.

**Additional file 3: Figure S3.** ODE induces minimal evidence of arthritis with or without CIA. **A)** Arthritis inflammatory score increases in HLA-DR4 transgenic mice following 4 weeks of ODE exposure. **B)** In the setting of arthritis induction (CIA), ODE-induced arthritis inflammatory score increases in WT-CIA + ODE and DR4-CIA + ODE animals. Statistical difference versus saline-treated control mice is indicated by #p < 0.05.

**Additional file 4: Figure S4**. Co-localization of MAA, CIT, and vimentin in the setting of arthritis induction (CIA) with and without ODE. CIT- and MAA-modified proteins, CIT, and vimentin, and MAA and vimentin strongly co-localize in all treatment groups. Statistical difference (####p < 0.0001) versus saline.

## References

[1] Kelly CA, Saravanan V, Nisar M, Arthanari S, Woodhead FA, Price-Forbes AN, Dawson J, Sathi N, Ahmad Y, Koduri G (2014). Rheumatoid arthritis-related interstitial lung disease: associations, prognostic factors and physiological and radiological characteristics—a large multicentre UK study. Rheumatology.

[2] England BR, Sayles H, Michaud K, Caplan L, Davis LA, Cannon GW, Sauer BC, Solow EB, Reimold AM, Kerr GS, Schwab P, Baker JF, Mikuls TR (2016). Cause-specific mortality in male US veterans with rheumatoid arthritis. Arthritis Care Res.

[3] England BR, Baker JF, Sayles H, Michaud K, Caplan L, Davis LA, Cannon GW, Sauer BC, Solow EB, Reimold AM, Kerr GS, Mikuls TR (2018). Body mass index, weight loss, and cause-specific mortality in rheumatoid arthritis. Arthritis Care Res.

[4] Gregersen PK, Silver J, Winchester RJ (1987). The shared epitope hypothesis. An approach to understanding the molecular genetics of susceptibility to rheumatoid arthritis. Arthritis Rheum.

[5] Ebel AV, Lutt G, Poole JA, Thiele GM, Baker JF, Cannon GW, Gaffo A, Kerr GS, Reimold A, Schwab P, Singh N, Richards JS, Ascherman DP, Mikuls TR, England BR (2021). Association of agricultural, occupational, and military inhalants with autoantibodies and disease features in US veterans with rheumatoid arthritis. Arthritis Rheumatol.

[6] Becart S, Whittington KB, Prislovsky A, Rao NL, Rosloniec EF (2021). The role of posttranslational modifications in generating neo-epitopes that bind to rheumatoid arthritis-associated HLA-DR alleles and promote autoimmune T cell responses. PLoS ONE.

[7] Cojocaru M, Cojocaru IM, Silosi I, Vrabie CD, Tanasescu R (2010). Extra-articular manifestations in rheumatoid arthritis. Maedica.

[8] Mori S, Koga Y, Sugimoto M (2012). Different risk factors between interstitial lung disease and airway disease in rheumatoid arthritis. Respir Med.

[9] Radoux V, Menard HA, Begin R, Decary F, Koopman WJ (1987). Airways disease in rheumatoid arthritis patients. One element of a general exocrine dysfunction. Arthritis Rheum.

[10] Oka S, Furukawa H, Shimada K, Sugii S, Hashimoto A, Komiya A, Fukui N, Suda A, Tsunoda S, Ito S, Katayama M, Nakamura T, Saisho K, Sano H, Migita K, Nagaoka S, Tsuchiya N, Tohma S (2016). Association of human leukocyte antigen alleles with chronic lung diseases in rheumatoid arthritis. Rheumatology.

[11] Grunewald J, Brynedal B, Darlington P, Nisell M, Cederlund K, Hillert J, Eklund A (2010). Different HLA-DRB1 allele distributions in distinct clinical subgroups of sarcoidosis patients. Respir Res.

[12] Grunewald J, Eklund A (2009). Lofgren's syndrome: human leukocyte antigen strongly influences the disease course. Am J Respir Crit Care Med.

[13] Sato H, Woodhead FA, Ahmad T, Grutters JC, Spagnolo P, van den Bosch JM, Maier LA, Newman LS, Nagai S, Izumi T, Wells AU, du Bois RM, Welsh KI (2010). Sarcoidosis HLA class II genotyping distinguishes differences of clinical phenotype across ethnic groups. Hum Mol Genet.

[14] Boyton RJ, Smith J, Jones M, Reynolds C, Ozerovitch L, Chaudhry A, Wilson R, Rose M, Altmann DM (2008). Human leucocyte antigen class II association in idiopathic bronchiectasis, a disease of chronic lung infection, implicates a role for adaptive immunity. Clin Exp Immunol.

[15] Sweatman MC, Markwick JR, Charles PJ, Jones SE, Prior JM, Maini RN, Turner-Warwick ME (1986). Histocompatibility antigens in adult obliterative bronchiolitis with or without rheumatoid arthritis. Dis Markers.

[16] Murphy D, Hutchinson D (2017). Is male rheumatoid arthritis an occupational disease? A review. Open Rheumatol J.

[17] Too CL, Muhamad NA, Ilar A, Padyukov L, Alfredsson L, Klareskog L, Murad S, Bengtsson C, My ESG (2016). Occupational exposure to textile dust increases the risk of rheumatoid arthritis: results from a Malaysian population-based case-control study. Ann Rheum Dis.

[18] Stolt P, Yahya A, Bengtsson C, Kallberg H, Ronnelid J, Lundberg I, Klareskog L, Alfredsson L, Group ES (2010). Silica exposure among male current smokers is associated with a high risk of developing ACPA-positive rheumatoid arthritis. Anna Rheum Dis.

[19] Stolt P, Källberg H, Lundberg I, Sjögren B, Klareskog L, Alfredsson L, Group ES (2005). Silica exposure is associated with increased risk of developing rheumatoid arthritis: results from the Swedish EIRA study. Ann Rheum Dis.

[20] Yahya A, Bengtsson C, Larsson P, Too CL, Mustafa AN, Abdullah NA, Muhamad NA, Klareskog L, Murad S, Alfredsson L (2014). Silica exposure is associated with an increased risk of developing ACPA-positive rheumatoid arthritis in an Asian population: evidence from the Malaysian MyEIRA case-control study. Mod Rheumatol.

[21] Rangel-Moreno J, Hartson L, Navarro C, Gaxiola M, Selman M, Randall TD (2006). Inducible bronchus-associated lymphoid tissue (iBALT) in patients with pulmonary complications of rheumatoid arthritis. J Clin Investig.

[22] Gizinski AM, Mascolo M, Loucks JL, Kervitsky A, Meehan RT, Brown KK, Holers VM, Deane KD (2009). Rheumatoid arthritis (RA)-specific autoantibodies in patients with interstitial lung disease and absence of clinically apparent articular RA. Clin Rheumatol.

[23] Janssen KM, de Smit MJ, Brouwer E, de Kok FA, Kraan J, Altenburg J, Verheul MK, Trouw LA, van Winkelhoff AJ, Vissink A, Westra J (2015). Rheumatoid arthritis-associated autoantibodies in non-rheumatoid arthritis patients with mucosal inflammation: a case-control study. Arthr Res Ther.

[24] Quirke AM, Perry E, Cartwright A, Kelly C, De Soyza A, Eggleton P, Hutchinson D, Venables PJ (2015). Bronchiectasis is a model for chronic bacterial infection inducing autoimmunity in rheumatoid arthritis. Arthr Rheumatol.

[25] Makrygiannakis D, Hermansson M, Ulfgren AK, Nicholas AP, Zendman AJ, Eklund A, Grunewald J, Skold CM, Klareskog L, Catrina AI (2008). Smoking increases peptidylarginine deiminase 2 enzyme expression in human lungs and increases citrullination in BAL cells. Ann Rheum Dis.

[26] Dogan H, Akgun M, Araz O, Ucar EY, Yoruk O, Diyarbakir E, Atis O, Akdemir F, Acemoglu H, Pirim I (2014). The association of human leukocyte antigen polymorphisms with disease severity and latency period in patients with silicosis. Multidiscip Respir Med.

[27] Vassallo R, Luckey D, Behrens M, Madden B, Luthra H, David C, Taneja V (2014). Cellular and humoral immunity in arthritis are profoundly influenced by the interaction between cigarette smoke effects and host HLA-DR and DQ genes. Clin Immunol.

[28] Xiong L, Xiong L, Ye H, Ma WL (2021). Animal models of rheumatoid arthritis-associated interstitial lung disease. Immun Inflamm Dis.

[29] Poole JA, Thiele GM, Janike K, Nelson AJ, Duryee MJ, Rentfro K, England BR, Romberger DJ, Carrington JM, Wang D, Swanson BJ, Klassen LW, Mikuls TR (2019). Combined collagen-induced arthritis and organic dust-induced airway inflammation to model inflammatory lung disease in rheumatoid arthritis. J Bone Miner Res.

[30] Pan S, Trejo T, Hansen J, Smart M, David CS (1998). HLA-DR4 (DRB1*0401) transgenic mice expressing an altered CD4-binding site: specificity and magnitude of DR4-restricted T cell response. J Immunol.

[31] Nelson AJ, Roy SK, Warren K, Janike K, Thiele GM, Mikuls TR, Romberger DJ, Wang D, Swanson B, Poole JA (2018). Sex differences impact the lung-bone inflammatory response to repetitive inhalant lipopolysaccharide exposures in mice. J Immunotoxicol.

[32] Poole JA, Dooley GP, Saito R, Burrell AM, Bailey KL, Romberger DJ, Mehaffy J, Reynolds SJ (2010). Muramic acid, endotoxin, 3-hydroxy fatty acids, and ergosterol content explain monocyte and epithelial cell inflammatory responses to agricultural dusts. J Toxicol Environ Health Part A.

[33] Poole JA, Wyatt TA, Oldenburg PJ, Elliott MK, West WW, Sisson JH, Von Essen SG, Romberger DJ (2009). Intranasal organic dust exposure-induced airway adaptation response marked by persistent lung inflammation and pathology in mice. Am J Physiol Lung Cell Mol Physiol.

[34] Mikuls TR, Gaurav R, Thiele GM, England BR, Wolfe MG, Shaw BP, Bailey KL, Wyatt TA, Nelson AJ, Duryee MJ, Hunter CD, Wang D, Romberger DJ, Ascherman DP, Poole JA (2021). The impact of airborne endotoxin exposure on rheumatoid arthritis-related joint damage, autoantigen expression, autoimmunity, and lung disease. Int Immunopharmacol.

[35] Gaurav R, Mikuls TR, Thiele GM, Nelson AJ, Niu M, Guda C, Eudy JD, Barry AE, Wyatt TA, Romberger DJ, Duryee MJ, England BR, Poole JA (2021). High-throughput analysis of lung immune cells in a combined murine model of agriculture dust-triggered airway inflammation with rheumatoid arthritis. PLoS ONE.

[36] Mikuls TR, Duryee MJ, Rahman R, Anderson DR, Sayles HR, Hollins A, Michaud K, Wolfe F, Thiele GE, Sokolove J, Robinson WH, Lingampalli N, Nicholas AP, Talmon GA, Su K, Zimmerman MC, Klassen LW, Thiele GM (2017). Enrichment of malondialdehyde-acetaldehyde antibody in the rheumatoid arthritis joint. Rheumatology.

[37] Thiele GM, Duryee MJ, Hunter CD, England BR, Fletcher BS, Daubach EC, Pospisil TP, Klassen LW, Mikuls TR (2020). Immunogenic and inflammatory responses to citrullinated proteins are enhanced following modification with malondialdehyde-acetaldehyde adducts. Int Immunopharmacol.

[38] Thiele GM, Duryee MJ, Anderson DR, Klassen LW, Mohring SM, Young KA, Benissan-Messan D, Sayles H, Dusad A, Hunter CD, Sokolove J, Robinson WH, O'Dell JR, Nicholas AP, Tuma DJ, Mikuls TR (2015). Malondialdehyde-acetaldehyde adducts and anti-malondialdehyde-acetaldehyde antibodies in rheumatoid arthritis. Arthr Rheumatol.

[39] Poole JA, Mikuls TR, Duryee MJ, Warren KJ, Wyatt TA, Nelson AJ, Romberger DJ, West WW, Thiele GM (2017). A role for B cells in organic dust induced lung inflammation. Respir Res.

[40] Ghosn EE, Yang Y, Tung J, Herzenberg LA, Herzenberg LA (2008). CD11b expression distinguishes sequential stages of peritoneal B-1 development. Proc Natl Acad Sci USA.

[41] England BR, Duryee MJ, Roul P, Mahajan TD, Singh N, Poole JA, Ascherman DP, Caplan L, Demoruelle MK, Deane KD, Klassen LW, Thiele GM, Mikuls TR (2019). Malondialdehyde-acetaldehyde adducts and antibody responses in rheumatoid arthritis-interstitial lung disease. Arthr Rheumatol.

[42] Gaurav R, Anderson DR, Radio SJ, Bailey KL, England BR, Mikuls TR, Thiele GM, Strah HM, Romberger DJ, Wyatt TA, Dickinson JD, Duryee MJ, Katafiasz DM, Nelson AJ, Poole JA (2021). IL-33 depletion in COVID-19 lungs. Chest.

[43] Inglis JJ, Simelyte E, McCann FE, Criado G, Williams RO (2008). Protocol for the induction of arthritis in C57BL/6 mice. Nat Protoc.

[44] Szeliga J, Hess H, Rude E, Schmitt E, Germann T (1996). IL-12 promotes cellular but not humoral type II collagen-specific Th 1-type responses in C57BL/6 and B10.Q mice and fails to induce arthritis. Int Immunol.

[45] Schurgers E, Billiau A, Matthys P (2011). Collagen-induced arthritis as an animal model for rheumatoid arthritis: focus on interferon-gamma. J Interferon Cytokine Res.

[46] Kato A, Hulse KE, Tan BK, Schleimer RP (2013). B-lymphocyte lineage cells and the respiratory system. J Allergy Clin Immunol.

[47] Perry E, Kelly C, Eggleton P, De Soyza A, Hutchinson D (2014). The lung in ACPA-positive rheumatoid arthritis: an initiating site of injury?. Rheumatology.

[48] Perry E, Eggleton P, De Soyza A, Hutchinson D, Kelly C (2017). Increased disease activity, severity and autoantibody positivity in rheumatoid arthritis patients with co-existent bronchiectasis. Int J Rheum Dis.

[49] Wysocki T, Olesinska M, Paradowska-Gorycka A (2020). Current understanding of an emerging role of HLA-DRB1 gene in rheumatoid arthritis-from research to clinical practice. Cells.

[50] Natalini JG, Baker JF, Singh N, Mahajan TD, Roul P, Thiele GM, Sauer BC, Johnson CR, Kawut SM, Mikuls TR, England BR (2021). Autoantibody seropositivity and risk for interstitial lung disease in a prospective male-predominant rheumatoid arthritis cohort of U.S. veterans. Ann Am Thorac Soc.

[51] Tan LK, Too CL, Diaz-Gallo LM, Wahinuddin S, Lau IS, Heselynn H, Nor-Shuhaila S, Gun SC, Eashwary M, Mohd-Shahrir MS, Ainon MM, Azmillah R, Muhaini O, Shahnaz M, Alfredsson L, Klareskog L, Padyukov L (2021). The spectrum of association in HLA region with rheumatoid arthritis in a diverse Asian population: evidence from the MyEIRA case-control study. Arthritis Res Ther.

[52] Werner J, Rivera N, Grunewald J, Eklund A, Iseda T, Darlington P, Kullberg S (2021). HLA-DRB1 alleles associate with hypercalcemia in sarcoidosis. Respir Med.

[53] Lard LR, Boers M, Verhoeven A, Vos K, Visser H, Hazes JM, Zwinderman AH, Schreuder GM, Breedveld FC, De Vries RR, van der Linden S, Zanelli E, Huizinga TW (2002). Early and aggressive treatment of rheumatoid arthritis patients affects the association of HLA class II antigens with progression of joint damage. Arthr Rheum.

[54] O'Dell JR, Nepom BS, Haire C, Gersuk VH, Gaur L, Moore GF, Drymalski W, Palmer W, Eckhoff PJ, Klassen LW, Wees S, Thiele G, Nepom GT (1998). HLA-DRB1 typing in rheumatoid arthritis: predicting response to specific treatments. Ann Rheum Dis.

[55] Sparks JA, Chang SC, Liao KP, Lu B, Fine AR, Solomon DH, Costenbader KH, Karlson EW (2016). Rheumatoid arthritis and mortality among women during 36 years of prospective follow-up: results from the nurses' health study. Arthr Care Res.

[56] Shaw M, Collins BF, Ho LA, Raghu G (2015). Rheumatoid arthritis-associated lung disease. Eur Respir Rev.

[57] Koduri G, Norton S, Young A, Cox N, Davies P, Devlin J, Dixey J, Gough A, Prouse P, Winfield J, Williams P, Eras (2010). Interstitial lung disease has a poor prognosis in rheumatoid arthritis: results from an inception cohort. Rheumatology.

[58] Olson AL, Swigris JJ, Sprunger DB, Fischer A, Fernandez-Perez ER, Solomon J, Murphy J, Cohen M, Raghu G, Brown KK (2011). Rheumatoid arthritis-interstitial lung disease-associated mortality. Am J Respir Crit Care Med.

[59] Sparks JA, Lin TC, Camargo CA, Barbhaiya M, Tedeschi SK, Costenbader KH, Raby BA, Choi HK, Karlson EW (2018). Rheumatoid arthritis and risk of chronic obstructive pulmonary disease or asthma among women: a marginal structural model analysis in the Nurses' Health Study. Semin Arthr Rheum.

[60] Nannini C, Medina-Velasquez YF, Achenbach SJ, Crowson CS, Ryu JH, Vassallo R, Gabriel SE, Matteson EL, Bongartz T (2013). Incidence and mortality of obstructive lung disease in rheumatoid arthritis: a population-based study. Arthr Care Res.

[61] Kelly KJ, Poole JA (2019). Pollutants in the workplace: Effect on occupational asthma. J Allergy Clin Immunol.

[62] Hoppin JA, Umbach DM, Long S, Rinsky JL, Henneberger PK, Salo PM, Zeldin DC, London SJ, Alavanja MC, Blair A, Beane Freeman LE, Sandler DP (2014). Respiratory disease in United States farmers. Occup Environ Med.

[63] Viatte S, Plant D, Han B, Fu B, Yarwood A, Thomson W, Symmons DP, Worthington J, Young A, Hyrich KL, Morgan AW, Wilson AG, Isaacs JD, Raychaudhuri S, Barton A (2015). Association of HLA-DRB1 haplotypes with rheumatoid arthritis severity, mortality, and treatment response. JAMA.

[64] Sapkota M, Burnham EL, DeVasure JM, Sweeter JM, Hunter CD, Duryee MJ, Klassen LW, Kharbanda KK, Sisson JH, Thiele GM, Wyatt TA (2017). Malondialdehyde-acetaldehyde (MAA) protein adducts are found exclusively in the lungs of smokers with alcohol use disorders and are associated with systemic anti-MAA antibodies. Alcohol Clin Exp Res.

[65] Li FJ, Surolia R, Li H, Wang Z, Liu G, Kulkarni T, Massicano AVF, Mobley JA, Mondal S, de Andrade JA, Coonrod SA, Thompson PR, Wille K, Lapi SE, Athar M, Thannickal VJ, Carter AB, Antony VB (2021). Citrullinated vimentin mediates development and progression of lung fibrosis. Sci Transl Med.

[66] Behrens M, Smart M, Luckey D, Luthra H, Taneja V (2011). To B or not to B: role of B cells in pathogenesis of arthritis in HLA transgenic mice. J Autoimmun.

[67] van Drongelen V, Scavuzzi BM, Nogueira SV, Miller FW, Sawalha AH, Holoshitz J (2021). HLA-DRB1 allelic epitopes that associate with autoimmune disease risk or protection activate reciprocal macrophage polarization. Sci Rep.

