# Supplementary figures and images for: Increased susceptibility to organic dust exposure-induced inflammatory lung disease with enhanced rheumatoid arthritis-associated autoantigen expression in HLA-DR4 transgenic mice
Source: Respir Res. 2022 Jun 18;23:160. doi: 10.1186/s12931-022-02085-8 (PMC9206339; doi:10.1186/s12931-022-02085-8)

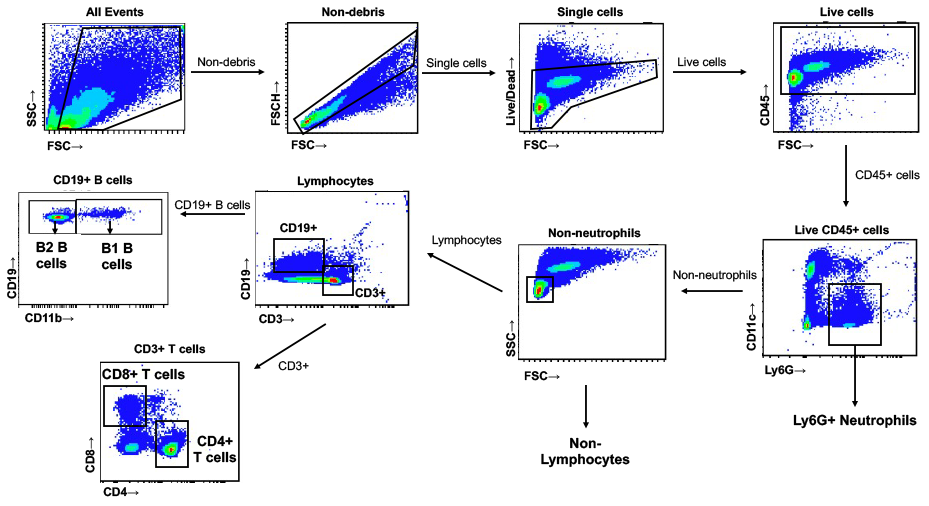

Supplement: Supplementary file 1 — Additional file 1: Figure S1. Flow cytometry gating strategies depicted for lung cell identification of Ly6G+ neutrophils, CD3+CD4+ T cells, CD3+CD8+ T cells, CD19+CD11b− B2 B cells and CD19+CD11b+ B1 B cells. [file 12931_2022_2085_MOESM1_ESM.tif]

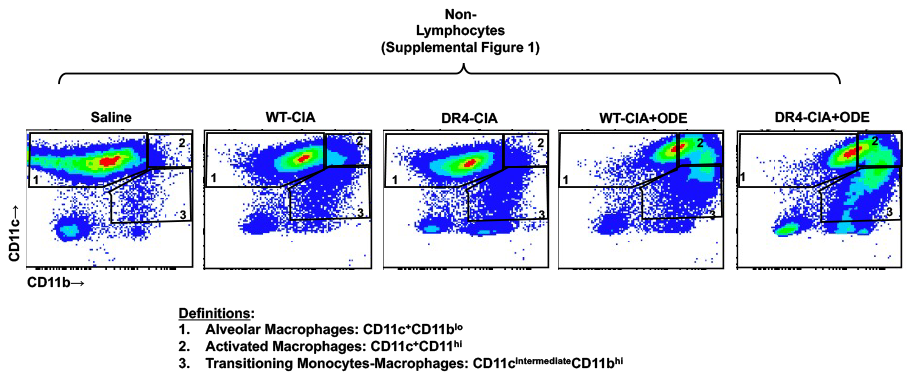

Supplement: Supplementary file 2 — Additional file 2: Figure S2. Flow cytometry gating strategy for lung macrophage-monocyte subpopulations is depicted. [file 12931_2022_2085_MOESM2_ESM.tif]

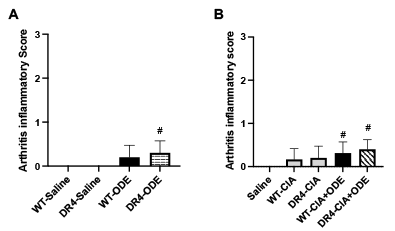

Supplement: Supplementary file 3 — Additional file 3: Figure S3. ODE induces minimal evidence of arthritis with or without CIA. A) Arthritis inflammatory score increases in HLA-DR4 transgenic mice following 4 weeks of ODE exposure. B) In the setting of arthritis induction (CIA), ODE-induced arthritis inflammatory score increases in WT-CIA + ODE and DR4-CIA + ODE animals. Statistical difference versus saline-treated control mice is indicated by #p < 0.05. [file 12931_2022_2085_MOESM3_ESM.tif]

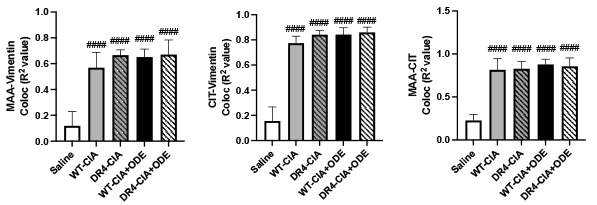

Supplement: Supplementary file 4 — Additional file 4: Figure S4. Co-localization of MAA, CIT, and vimentin in the setting of arthritis induction (CIA) with and without ODE. CIT- and MAA-modified proteins, CIT, and vimentin, and MAA and vimentin strongly co-localize in all treatment groups. Statistical difference (####p < 0.0001) versus saline. [file 12931_2022_2085_MOESM4_ESM.tif]
